# Feeding Behaviour and Nutritional Status among Children Aged 6 to 23 Months Old in Bahi District in Tanzania

**DOI:** 10.24248/eahrj.v8i2.779

**Published:** 2024-06-26

**Authors:** Leonida Tawa Chipanha, Leonard Katalambula

**Affiliations:** a University of Dodoma, College of Health science, Department of Nursing and Midwifery; b The University of Dodoma, School of Nursing and Public Health, Department of Public Health

## Abstract

**Background:**

The survival, well-being, and development of young children depend on optimal infant and young child feeding (IYCF) practices. It is imperative to assess nutrition status and feeding behaviour practices in order to develop interventions. The objective of this study is to assess nutrition status, feeding behaviour, and its association with nutrition status among children aged 6 to 23 months old in Bahi district, Tanzania.

**Methods:**

A community-based, cross-sectional study was employed. A multistage sampling technique was used. Bahi district council, wards, and village were randomly selected, and then a systematic random sampling method was used to select participants. Anthropometric measurements were used to determine the nutrition status of children. Process for the Promotion of Child Feeding (ProPAN) manual guided assessment of feeding behaviours associated with nutrition status. Z-score was used to determine the level of malnutrition; the chi-square test and logistic regression were used as descriptive and inferential statistical tests, respectively, to determine the association between nutrition status and feeding behaviour.

**Results:**

Out of 395 children aged between 6 and 23 months, 62.8% were stunted, 25.3% were underweight, and 6.6% were wasted. In the multivariable analysis, the results reveal that stunting was significantly associated with perception of exclusive breastfeeding (number of months) (AOR 4.24, 95%CI: 2.012–8.284) and number of feeds per day (AOR 2.02, 95%CI: 1.00–6.314). With regard to perception of exclusive breastfeeding (number of months), the children whose mothers perceived that children supposed to be breastfed for more than six months were four times more likely to be not stunted compared to those who were perceived to be breastfed less than three months, and the children whose mothers' fed them twice to three times were two times more likely to be not stunted compared to the children who were fed only two times.

**Conclusion:**

Bahi district council has a higher prevalence rate of stunting, underweight, and wasting among children aged 6 to 23 months. Feeding on the first colostrum, exclusive breastfeeding, and breastfeeding for more than six months are the main determinants of nutrition status.

## BACKGROUND

The high burden of malnutrition among children has contributed to the increased rate of child morbidity and mortality.^1^ Worldwide, approximately 21.9% of 149 million children under five were reported to suffer from malnutrition in 2018. In 2020, 22% of all children under five globally were malnourished, while severe wasting and overweight were 6.7% and 5.6%, respectively.^2^ Half of these children are from Asia, and one-third were from Africa. However, Africa has experienced a tremendous increase in malnutrition, from 50.3 million children in 2000 to 58.8 million children in 2018. In 2015, Sub Saharan Africa (SSA) contributed almost 30% of the global burden of under-five child malnutrition.^3^ This trend is not a good indicator of the global sustainable development goals (SDG3), which target reducing the number of under-five children with malnutrition by 50% and reducing both severe wasting and overweight to less than 3%.^4^ However, the WHO member countries committed to increasing their efforts to reduce under-5 child stunting to a target of less than 40% and severe wasting and overweight to 5% by 2025.^5^

Nutritional assessment during childhood is necessary in order to determine any abnormal nutritional disorders and take action on time by improving feeding behaviours.^6^ The childhood stage has recently been recognised as a potential target for preventing malnutrition disorders in adulthood, such as underweight and obesity.^7^ Feeding behaviour is a concern of many researchers since it has a direct influence on the child's growth and survival; hence, it is highly associated with many health outcomes^8^. Several studies and reports have shown feeding behaviour can affect children's nutritional status, which can impact across the continuum of malnutrition (i.e., stunting, wasting, underweight, and overweight). ^9,10,11^ The feeding behaviour may affect children's physical and cognitive development, weaken immunity, and increase the risk of death.^4^ Feeding practices are affected by a number of factors, including; feeding behaviour, complementary food feeding, and frequency of feeding. ^12^ Alleviating the challenges of nutritional status among children can potentially be addressed by improving feeding behaviour, including maternal nutrition, access to primary health care, breastfeeding practices, and child feeding, and strengthening the health care system.^13^ Moreover, feeding behaviours have a direct relationship with preventing or promoting undernutrition and are linked to deficiency diseases such as kwashiorkor, marasmus, vitamin A deficiency, anaemia, and goitre.^14^ Tanzania is among ten countries of Africa, reporting a high number of children with under-nutrition. Very minimal change has been observed with the decline between 2014 and 2022 being 34.7% to 30.% respectively.^15^ Shockingly, high under-nutrition rates in Tanzania are reported in food-producing regions, such as Iringa (57.%), Njombe (50.%), and Rukwa (50.%).^15^ This anomaly has been attributed to poor feeding behaviours which are influenced by social norms, cultural practices, and taboos, such as cultural beliefs in some tribes that babies should not consume colostrum.^16^

Tanzania is among the African countries reporting a high number of children with undernutrition, which is a consequence of feeding behaviour.^2^ Almost 40% of undernourished children in Tanzania are between 6 and 23 months of age. The national prevalence of stunting is 30%, while wasting and underweight are 3% and 12%, respectively.^17^ Tanzania has been implementing several nutritional programmes and interventions to reduce malnutrition in children under five. These interventions include deworming, mass distribution of vitamin A supplements, and the infant and young child feeding (IYCF) protocols.^15^ Dodoma region, where Bahi district is located, is one of the areas with the highest frequency and quantity of stunted children. Approximately 31% of children under the age of five in the Dodoma region are stunted overall, which is higher than the national average (DHS 2022).

Assessing feeding behaviour practices is essential for identifying issues with breastfeeding, complementary feeding, and early childhood nutrition. It also helps define the context in which these issues arise, including factors that support and impede the adoption of better or “ideal” practices. Therefore, this study was conducted to assess the feeding behaviours and their association with the nutritional status of children aged between 6 and 23 months in Bahi district.

## METHODS

### Study Area and Setting

A community-based cross-sectional survey was conducted in households among children aged 6–23 months in five villages of the Bahi district, which is among the seven districts of Dodoma Region. The district is located between latitudes 5° and 8° South and longitudes 35° and 37° East.^18^ The district shares a border with Singida region in the west and Chamwino district to the east. It borders Chemba in the north and Dodoma City in the west. According to the 2012 census of housing and population, Bahi District Council has a population of 258,620. However, the region reported the current population to be 134,518 women (of which 40,367 are of childbearing age) and 124,103 men. NBS shows that the district has 46,754 children less than five years of age. Roggie ^19^ reports that the district has an area of 5,631 square kilometres with a total of 20 wards and 59 villages served by 37 dispensaries and 6 health centres.

### Sample Size and Sampling Procedures

The sample size of 395 children aged 6–23 months was obtained by using Bartlett et al.'s formula.^20^ The multi-stage sampling technique was used to obtain the study participants. Five stages were involved. The first stage was the selection of the Dodoma region out of 10 regions in Tanzania with high rates of undernutrition by using a simple random selection method. The second stage was the selection of one district from eight using simple random techniques, whereby Bahi was selected. One ward out of twenty-two wards was selected using simple random techniques. And one village was selected by using simple random techniques. The fifth stage was the selection of the household's systematic, simple random selection techniques. In the event of the absence of the children, the replacement was done in the next household. Children with diseases and deformities Were excluded from the study.

### Data Collection Methods and Instruments

The data collection procedure was done by the principal researcher, assisted by trained health workers. The information collected included demographic characteristics, social and economic information, types of traditional or local methods for feeding practices for infants; coping strategies used to ensure frequency of breastfeeding, and patterns of diseases among infants.

A number of instruments were used throughout this study. A structured questionnaire was adopted from the ProPAN tool (WHO) checklist, with a length board with a moving head piece measuring height and an adjusted SECA scale used to measure weight. Additionally, the mid-upper arm circumference (MUAC) tape was used to obtain critical measurements, and Reproductive and Child Health (RCH) Card No. 4 was assessed for additional information such as date of birth and birth weight. The questionnaire was translated into Swahili and piloted prior to the field data collection. Parents of their children signed the consent, which was voluntary, before participating in the study.

Anthropometric data were collected by the trained research assistant, who also administered the questionnaire. Nutrition assessment was done using the length board with a moving head piece measuring the height, and an adjusted SECA scale was used to measure weight. Quality of the measurements was ensured by maintaining consistency of the anthropometric measurements. Data collectors were tested using ENA for smart software before starting the data collection.

### Data Management

The data were cleaned and analysed using the Statistical Package for Social Science (SPSS) Version 20. The data were first classified into meaningful categories to make analysis easy and viable, yielding descriptive statistics in the form of percentages and frequencies. A chi-square test tested the association of descriptive variables, and comparison was done by logistic regression, whereby OR and AOR were presented. Anthropometric data was analysed by the Emergency Nutrition Assessment (ENA) for smart and three indices—specifically, height for age, weight for height, and weight for age. Z-scores were determined to assess the nutrition status of infants as compared to the National Centre of Health Statistics (NCHS)/WHO reference standards.

Seven questions were used to measure the weight, height, MUAC, and age of the child, which were converted to Z-scores. The cut-off point of ≤2SD was LHAZ (Low Height for Age Z-scores), LHWZ (Low Height for Weight Z-score), LWAZ (Low Weight for Age Z-score) was defined as moderate under-nutrition, ≤3SD defined as severe under-nutrition >+2SD define HW= Overweight, weight with 95% confidence intervals (Table 1)

**Table 1.** Cut-Off Points for Definition of Wasting, Stunting and Underweight

Classification	Acute malnutrition or wasting (WHZ)	Acute malnutrition or stunting (HAZ)	Underweight (WAZ)
Global	≤ 2 SD &/or bilateral Oedema	≤ 2 SD	≤ 2 SD
Moderate	-2 SD & -3 SD	-2 SD & -3 SD	-2 SD & -3 SD
Severe	≤ -3 SD/or bilateral oedema	< -3 SD	< -3 SD

WAZ-Weight for Age Z-score, HAZ-Height for Age Z-scores, WHZ-Weight-Height Z-scores

## RESULTS

### Demographic Characteristics of Respondents

A total of 395 children aged 6 to 23 months and their mothers or carers, with most aged between 15 and 19 years old, or 173 (43.8%). Regarding the level of education, most mothers or carers (305, 77.2%) had no formal education and most participants (373, 94.4%) were married, while the main livelihood activity for most households (232, 58.7%). was through farming (Table 2).

**Table 2.** Demographic Characteristics of Mothers/Caregivers N =395

Variable	Frequency (n)	Percentage (%)
Age of mother		
15-19	173	43.8
20-35	174	44.1
36 and above	48	12.2
Education of mother		
No formal education	74	18.7
Primary	305	77.2
Secondary and above	16	4.1
Marital status of mother/caregiver		
Married	373	94.4
Unmarried	22	5.6
Genders of household head		
Mother	52	13.2
Father	343	86.8
People living in the home		
Two	16	4.1
Three	82	20.8
Four	55	13.8
Five	53	13.4
Six and above	189	47.9
Household's main source of income		
Salaried	88	22.3
Livestock keeping	59	14.9
Small business	16	4.1
Farmer	232	58.7
Caregiver's main occupation		
Livestock keeping	103	35.2
Small business	100	37.2
Farmer	186	26.1
Salaried	36	1.5

### Nutrition Status for Children Aged between 6-23 Months in Bahi Dodoma

Out of 395 children aged 6-23 months in the Bahi Dodoma at 95% CI (4.5–9.5), 6.6% were found to have wasting (Global Acute Malnutrition GAM), and at 95% CI (1.6–4.9), 2.8% of them suffered from severe acute malnutrition (SAM), while at 95% CI (57.9–67.4), 62.8% were stunted (Global Acute Malnutrition GAM), and among them at 95% CI (30.4–39.8), 34.9% were severe stunted (Severe Acute Malnutrition SAM). Also, at 95% CI (21.3–29.8), 25.3% were underweight (global acute malnutrition, GAM), and among them at 95% CI (5.4–10.6), 7.6% had severe acute malnutrition (SAM). (Table 3)

**Table 3.** Nutrition Status for Children Aged between 6-23 Months in Bahi District

	Wasting (%)			Stunting (%)			Underweight (%)		
	Boys	Girls	All	Boys	Girls	All	Boys	Girls	All
GAM	16 (6.8)	10 (6.3)	26 (6.6)	153 (61.7)	84 (53.1)	237 (62.8)	66 (28.1)	34 (21.3)	100 (25.3)
MAM	10 (4.3)	5 (3.1)	15 (3.8)	61 (26.0)	49 (30.6)	110 (27.8)	54 (23.0)	16 (10.0)	70 (17.7)
SAM	6 (2.6)	5 (3.1)	11 (2.8)	102 (28.1)	36 (21.3)	138 (25.3)	12 (5.1)	18 (11.3)	30 (7.6)

### Feeding Behaviour of Children Aged 6-23 Months among Households in Bahi district

With regards to the feeding behaviour of the 395 enrolled children, the majority (358, 90.6%) were fed with colostrum first, most (304, 77%) were breastfed more than nine times per day, and 139 (35.2%) of them believed breastfeeding for four to six months constituted exclusive breastfeeding. (Table 4)

**Table 4.** Feeding Behaviour of the Child (N=395)

Variable	Frequency	Percent
First child feed		
Colostrum	358	90.6
Other	37	9.40
Exclusively breastfed (6 months)		
Yes	139	35.2
No	256	64.8
Frequency of breastfeeds (per day)		
5 times	12	3.00
6 times	38	9.60
9 times	41	10.4
more than 9 times	304	77.0
Perception on exclusive breastfeeding (No. of months)		
< 6 months	302	76.5
≥ 6 months	92	23.3
Who feeds a child when you are not around		
House girl	25	6.30
Grandmother	217	54.9
Move with the child	26	6.60
Elder sister	127	32.2
First foods introduced		
Fluids	293	74.2
Very thin porridge	98	24.8
Semi-solid foods	4	1
Preparation of the first food introduced to the child		
Separately from the family meal	266	67.3
Together with the family meal	129	32.7
Number of feeds (per day)		
2 times	229	58.0
3 times	116	29.4
4-5 times	50	12.7

### Relationship Between Feeding Behaviour and Stunting (height for age)

Regarding the relationship between feeding behaviour and stunting, there was only a significant relationship between first thing fed and food introduced first with stunting, whereby the chi-square and *P* values were X^2^ =4.954 and X^2^ =7.228, with *P* =.026 and *P* =026, respectively. (Table 5)

**Table 5.** Relationship Between Feeding Behaviour and Stunting (Height for Age)

Variable	Height for Age		Chi-sq	*P-value*
	Stunted	not stunted		
First child feed				
Colostrum	231 (64.5%)	127 (35.5%)		
Other (non-colostrum)	17 (45.9%)	20 (54.1%)	4.954	.026
Child breastfeed with yellowish milk				
Yes	222 (64.3%)	123 (35.7%)		
No	26 (52%)	24 (48%)	2.85	.091
Exclusively breastfed				
Yes	87 (62.6%)	52 (37.4%)		
No	161 (62.9%)	95 (37.1%)	0.003	.953
Frequency of breastfeeds (per day)				
5 times	6 (50%)	6 (50%)		
6 times	20 (52%)	18 (48%)		
9 times	24 (58.5%)	17 (41.5%)		
more than 9 times	198 (65.1%)	106 (34.9%)	3.549	.314
Perception on exclusively breastfeeding (No. of the month)				
3 months	27 (67.5%)	13 (32.5%)		
4 months	80 (65%)	43 (35%)		
5-6 months	75 (53.9%)	64 (46.1%)		
more than 6 months	65 (70.6%)	27 (29.4%)	8.315	.081
Who feeds a child when you are not around				
House girl	19 (76%)	6 (24%)		
Grandmother	133 (61.3%)	84 (38.7%0		
Move with the child	12 (46.2%)	14 (53.8%)		
Elder sister	84 (66.1%)	43 (33.9%)	5.766	.124
First foods introduced				
Separately from the family meal	172 (64.7%)	94 (35.3%)		
Together with the family meal	76 (58.9%)	53 (41.1%)	1.228	.268
Number of feeds per day				
2 times	149 (65.1%)	80 (34.9%)		
2-3	69 (58%)	47 (42%)		
4-5 times	30 (60%)	20 (40%)	1.217	.544
Diversity in last 24-hour foods fed				
4-5 items (Diverse diet)	123 (66.1%)	63 (33.9%)		
2-3 items (non-diverse diet)	125 (59.8%)	84 (40.2%)	1.683	.195
Foods were introduced first				
Fluids	183 (62.4%)	110 (37.6%)		
Very thin porridge	65 (66.3%)	33 (33.7%)		
Semi-solid foods	0 (0%)	4 (100%)	7.288	.026

### Univariate and Multivariate Logistic Regression on Feeding Behaviour and Stunting (Height for Age)

Regarding the association between feeding behaviour and stunting, the results reveal that stunting was significantly associated with the perception of exclusive breastfeeding (number of months) (AOR 4.24, 95%CI: 2.012-8.284) and number of feeds per day (AOR 2.02, 95%CI: 1.003-6.314). About the perception of exclusive breastfeeding (number of months), the children whose mothers perceived that children were supposed to be breastfed for more than six months were four times more likely to be not stunted compared to those who were perceived to be breastfed less than three months, and the children whose mothers fed them twice to three times were two times more likely to be not stunted compared to the children who were fed only two times. (Table 6)

**Table 6.** Univariate and Multivariate Logistic Regression on Feeding Behaviour and Stunting (Height for Age)

Variables	Stunting status Stunted	Not stunted	Adjusted logistic model	
			OR (95%CI)**	AOR (95%CI)***
First child feed				
Other (non-colostrum)	231	127	ref	
Colostrum	17	20	1.379 (0.124-2.164)	1.68 (0.146-3.144)
Exclusively breastfed				
No	87	52	ref	
Yes	161	95	4.406 (0.178-6.925)	2.43 (0.112-4.225)
Frequency of Breastfeeds (per day)				
5 times	6	6	ref	
6 times	20	18	1.619 (0.069-2.342)	1.21 (0.379-5.562)
9 times	24	17	2.619 (0.161-2.383)	2.03 (0.365-2.383)
more than 9 times	198	106	3.021 (2.218, 6.097)	2.82 (0.348, 3.097)
Perception on exclusively breastfed (No. of months)				
3 months	27	13	ref	
4 months	80	43	1.254 (0.112-3.765)	1.24 (1.024-4.267)
5-6 months	75	64	2.354 (0.201-4.234)	2.00 (0.238-5.125)
more than 6 months	65	27	3.342 (0.223-6.365)	4.24 (2.012-8.284)
Who feeds the child when you are not around				
House girl	19	6	ref	
Grandmother	133	84	1.469 (0.044-4.963)	1.03 (0.224-2.934)
Move with the child	12	14	3.210 (0.06-4.736)	2.21 (0.163-4.425)
Elder sister	84	43	0.3 (0.046-1.981)	0.30 (0.046-1.981)
Foods were introduced first				
Fluids	183	110	ref	
Very thin porridge	65	33	1.12 (0.012-1.054)	1.05 (0.132-3.865)
Semi-solid foods	0	4	1.23 (0.12-2.043)	1.24 (0.108-4.234)
Separately from the family meal			1.859 (0.34-2.167)	2.90 (0.34-4.167)
Number of feeds (per day)				
2 times	149	80	ref	
3 times	69	47	2.961 (0.217-5263)	2.02 (1.003-6.314)
4-5 times	31	20	3.972 (0.202-5.68)	2.26 (0.08-5.674)

* p<0.05

** crude odds ratio with 95%CI

*** Adjusted odds with 95% CI

## DISCUSSION

The current study found the prevalence of stunting, underweight, and wasting among children aged between 6 and 23 months in the Bahi district council to be high compared to the regional and country averages.^15^ This difference may be caused by differences in weather conditions and cultural practices. Despite the fact that Bahi is a major producer of rice, a large area is dry with semi-arid characteristics. Bahi District has a short-wet season from January to March, followed by a long dry period between April and early December per year. The average rainfall is 500–800 mm per year. Rainfall is not only somehow low but also changeable in frequency and amount. It is this unreliable rainfall that has imposed risk in traditional agriculture and that represents a serious constraint on efforts to improve crop yields. The findings of this study are comparable to those conducted in North East Ethiopia and Western Kenya.^21^ Similarities between the two studies might be due to the same weather conditions and cultural practices of the community. This study's results are comparable to those of the current study, which may be explained by the similarities in cultural customs and economic circumstances between Tanzania and Kenya, both of which are low- and middle-income nations. In line with the present study, the previous study conducted in Bule Hora district in South Ethiopia among children aged between 6 and 59 months old found a high stunting rate, a relatively high underweight rate, and a low rate of wasting.^11^ The present study found a low rate of Severe Acute Malnutrition among children aged between 6 to 23 months. A study conducted in Northern India which has also found a low rate of Severe Acute Malnutrition among children aged between 6 months to 5 years^.22^ This study varies with the study conducted in an emergency paediatric unit at tertiary centre in North Central Nigeria which founds high rate of Severe Acute Malnutrition among admitted children aged between 6 to 60 months.^23^ The reason for the discrepancies could be the fact that whilst other studies were carried out in the community by visiting homes, this one was done on sick youngsters.

A recent study found high rates of global acute malnutrition compared to the current average among children aged between 6 and 23 months old. Likewise, the study conducted in North West Uganda found a high rate of global acute malnutrition among children aged between 6 and 59 months.^24^ This is due to the fact that these studies were conducted in a certain part of the country, where some parts have a high global acute malnutrition rate while others have a low rate, which is influenced by several factors, including weather conditions, cultural practices, and traditional practices.

In Bahi Dodoma, children between the ages of 6 and 23 months had a high risk of moderately severe malnutrition, according to the current study. This is consistent with research that showed a high rate of moderate to severe malnutrition in children under five in the war-torn Lake States of southern Sudan. The present study found a high rate of moderate-to-severe malnutrition among children aged between 6 and 23 months in Bahi Dodoma. This is in line with the study conducted in the War-Torn Lake States of Southern Sudan, which found a high rate of moderate-to-severe malnutrition among under-five children.^25^ The similarities might be due to factors such as feeding practices, food availability, and community cultural and traditional practices.

The current study found that the feeding behaviours most practiced by mothers with children aged between 6 and 23 months were breastfeeding with colostrum, yellowish milk, and a frequency of breastfeeding of more than 9 times per day. In comparison, a study conducted in Western Kenya found that history of diarrhoea, first food given during the first 6 months of life, and accessibility of food were predictors of wasting. ^21^ Likewise, a study conducted in Hora, South Ethiopia, with children aged between six and nine months found that the presence of diarrhoea in the past two weeks, pre-lacteal age at the time when complementary feeding was started, and the use of family planning methods were the predictors for the wasting of children.^11^ In contrast, a study conducted in Southern Benin found that social and economic aspects, family and cultural practices, as well as geographical and household demographics, influenced feeding practices.^26^

The present study found that the feeding behaviour factors influencing nutritional status in terms of underweight were the first thing fed to the child, feeding of yellowish milk, frequency of breastfeeding, perceptions of exclusive breastfeeding, and number of feedings per day. In contrast, a study conducted in Ghana among under-five-year-old children found age, wealth status, maternal education, region, ethnicity, household toilet facility, source of drinking water, incidence of diarrhoea, and subscription to health insurance were determining factors for underweight children.^27^ Likewise, a study conducted in Ethiopia among children aged between six and fifty-nine months found maternal education, family size, and diarrhoea morbidity in the past 12 months were associated with underweight children.^9^

The current study found that the feeding behaviour factors influencing nutritional status in terms of stunting were the perception of exclusive breastfeeding and the number of times a child feeds per day. A study conducted in Southwest Uganda among children aged between six and fifty-nine months old found that introducing complementary feeding at the appropriate age, primary carers knowledge about stunting, and food insecurity in the household were associated with stunting of a child.^28^ Similarly, a study conducted in Zuria, South Ethiopia, among children between six and nine months old found that pre-lacteal feeding, diarrhoea morbidity, household income, being female, and mothers who do not use family planning were predictors of childhood stunting.^10^

## CONCLUSION

Bahi district council has a higher prevalence rate of stunting, underweight, and wasting among children aged 6 to 23 months. Feeding on the first colostrum, exclusive breastfeeding, and breastfeeding for more than six months are the main determinants of nutrition status. This highlights the need to provide targeted infant and young child feeding education to mothers during antenatal and postnatal clinics.

### Study Limitation

The findings of this study may be influenced by the cross-sectional nature of the quantitative approach without complementing other approaches. The study also assessed children aged 6–23 months, whereas most studies focus on children under five.
